# Roles of Raft-Anchored Adaptor Cbp/PAG1 in Spatial Regulation of c-Src Kinase

**DOI:** 10.1371/journal.pone.0093470

**Published:** 2014-03-27

**Authors:** Takashi Saitou, Kentaro Kajiwara, Chitose Oneyama, Takashi Suzuki, Masato Okada

**Affiliations:** 1 Department of Molecular Medicine for Pathogenesis, Graduate School of Medicine, Ehime University, Shitsukawa, Toon, Ehime, Japan; 2 Department of Oncogene Research, Research Institute for Microbial Diseases, Osaka University, Suita, Osaka, Japan; 3 Division of Mathematical Science, Department of Systems Innovation, Graduate School of Engineering Science, Osaka University, Toyonaka, Osaka, Japan; 4 JST, CREST, Chiyoda-ku, Tokyo, Japan; Boston University School of Medicine, United States of America

## Abstract

The tyrosine kinase c-Src is upregulated in numerous human cancers, implying a role for c-Src in cancer progression. Previously, we have shown that sequestration of activated c-Src into lipid rafts via a transmembrane adaptor, Cbp/PAG1, efficiently suppresses c-Src-induced cell transformation in Csk-deficient cells, suggesting that the transforming activity of c-Src is spatially regulated via Cbp in lipid rafts. To dissect the molecular mechanisms of the Cbp-mediated regulation of c-Src, a combined analysis was performed that included mathematical modeling and *in vitro* experiments in a c-Src- or Cbp-inducible system. c-Src activity was first determined as a function of c-Src or Cbp levels, using focal adhesion kinase (FAK) as a crucial c-Src substrate. Based on these experimental data, two mathematical models were constructed, the sequestration model and the ternary model. The computational analysis showed that both models supported our proposal that raft localization of Cbp is crucial for the suppression of c-Src function, but the ternary model, which includes a ternary complex consisting of Cbp, c-Src, and FAK, also predicted that c-Src function is dependent on the lipid-raft volume. Experimental analysis revealed that c-Src activity is elevated when lipid rafts are disrupted and the ternary complex forms in non-raft membranes, indicating that the ternary model accurately represents the system. Moreover, the ternary model predicted that, if Cbp enhances the interaction between c-Src and FAK, Cbp could promote c-Src function when lipid rafts are disrupted. These findings underscore the crucial role of lipid rafts in the Cbp-mediated negative regulation of c-Src-transforming activity, and explain the positive role of Cbp in c-Src regulation under particular conditions where lipid rafts are perturbed.

## Introduction

**Figure 1:**
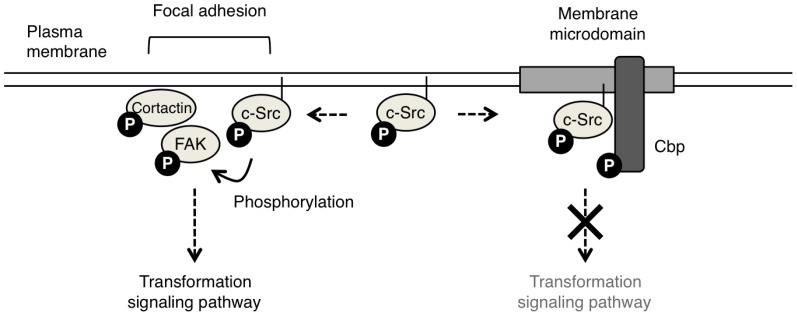
Proposed hypothesis for the spatial control of c-Src phosphorylation. c-Src is anchored to the plasma membrane via its N-terminal myristoyl modification. In focal adhesions, activated c-Src phosphorylates its substrates, FAK and cortactin. When Cbp is expressed, activated c-Src is retained in lipid rafts by Cbp. This results in the sequestration of c-Src from Src substrates, thereby suppressing the phosphorylation of Src substrates.

The first identified proto-oncogene product, c-Src [1], is a membrane-associated non-receptor tyrosine kinase that plays pivotal roles in coordinating a broad range of cellular responses such as differentiation, proliferation, adhesion, and migration [2]. The kinase activity of c-Src is enhanced by autophosphorylation at tyrosine 418 (Y418) in response to extracellular stimuli such as growth factors and extracellular matrices, while c-Src activity is negatively regulated by phosphorylation of its regulatory tyrosine 529 (Y529), which is catalyzed by the C-terminal Src kinase (Csk) [3]–[5]. The regulatory mechanism for c-Src function has been extensively analyzed by molecular studies [6] as well as theoretical studies [7]–[10], but c-Src signaling dynamics and their roles in cell physiology and diseases such as cancer are not yet fully understood.

c-Src is frequently overexpressed and activated in a wide variety of human cancers [6], [11], [12], despite the fact that Csk is normally expressed and the *c-src* gene is not mutated [13], [14]. These observations suggest that other components in the c-Src regulatory system may be perturbed during cancer progression, although the underlying mechanisms remain unclear. Upregulation of c-Src has been implicated in cancer invasion and metastasis, which are associated with the activation of cell-migration machinery [15]. Cell migration is mediated by the formation and disassembly of focal adhesions [16], which is controlled by c-Src-mediated phosphorylation of focal adhesion components such as focal adhesion kinase (FAK) and cortactin [17], [18]. Constitutive activation of FAK promotes not only focal adhesion turnover, but also cell growth and survival signaling, thereby promoting tumor progression [16]. Upon activation of c-Src, c-Src and FAK tightly interact to phosphorylate and activate each other, but the mechanism through which activated c-Src efficiently accesses FAK remains elusive.

c-Src is anchored to the membrane via its myristoylated N-terminus, whereas Csk is a cytoplasmic protein; thus Csk requires membrane-anchor proteins to efficiently access c-Src. A transmembrane phosphoprotein, Csk-binding protein (Cbp) [19], also called PAG1 [20] but hereafter referred to as Cbp, has been identified as such a membrane anchor for Csk. Cbp is exclusively localized to lipid rafts, membrane microdomains enriched in cholesterol and sphingolipids [21], by palmitoylation anchoring. Lipid rafts have been regarded as signaling platforms that harbor various signaling molecules and positively transduce cell signaling, although the specific function of lipid rafts is still under debate [22]. In normal cells, Cbp in lipid rafts plays a scaffolding role in the Csk-dependent negative regulation of c-Src [19], [23]. We analyzed the role of Cbp in the regulation of the transforming activity of c-Src using Csk-deficient cells as a model system [24]. This system enabled us to dissect the initial events following c-Src activation, and to mimic the activity status of c-Src in cancer cells, in which the proportion of c-Src present in the active form is increased despite the expression of Csk [24], [25]. Because Csk-deficient cells can be transformed by expression of a limited amount of wild-type (WT) c-Src, the cells were also used to identify c-Src targets required for cell transformation. In this system, we found that phosphorylated Cbp binds to c-Src via its Src homology 2 (SH2) domain and recruits active c-Src to lipid rafts, and that sequestration of c-Src in lipid rafts is sufficient to suppress c-Src-mediated transformation (Figure 1) [25], [26]. The tumor-suppressive role of Cbp was verified in v-Src-transformed cells and in human cancer cells, both of which express Csk [26].

We also showed that the distribution of Src family kinases (SFKs) to lipid rafts varies, depending upon their N-terminal fatty-acylation status [26]. c-Src and Blk, which have a single myristoyl moiety, have a low affinity for lipid rafts and mostly distribute to non-raft membranes when Cbp is downregulated. By contrast, other SFK members, such as Lyn and Fyn, which contain additional palmitoyl moieties, have a higher affinity for lipid rafts, and can therefore distribute to lipid rafts independently of Cbp. It was reported that Cbp could function positively in SFK-mediated signal transduction when bound to raft-localized SFKs, such as Lyn and Fyn, in lipid rafts [27]–[29]. These findings suggest that Cbp positively supports the function of SFKs that are intrinsically localized in lipid rafts, while it negatively regulates non-raft c-Src/Blk by spatially controlling their raft localization. However, the mechanisms by which Cbp exerts such reciprocal functions remain unknown.

In this study, we addressed the molecular mechanisms for the Cbp-mediated spatial regulation of the transforming activity of c-Src. For this purpose, a mathematical modeling analysis was combined with *in vitro* experiments using a c-Src or Cbp inducible system in Csk-deficient cells. This system is suitable for analyzing the initial processes in cell transformation or its suppression [30]. The combined analysis reveals that in addition to Cbp-Src and Src-FAK complexes, a ternary complex consisting of Cbp, c-Src and FAK is required for the Cbp-mediated regulation of c-Src via lipid rafts. Moreover, the proposed model predicts that, if Cbp enhances the interaction between c-Src and FAK, Cbp can further promote c-Src function when lipid rafts are perturbed. These findings underscore the crucial role of lipid rafts in the Cbp-mediated regulation of c-Src, and suggest that Cbp can positively regulate c-Src function under particular conditions, such as where lipid rafts are perturbed or c-Src substrates are present in lipid rafts.

## Results

### *In vitro* analysis of c-Src activity regulated by Cbp and lipid rafts

To analyze the regulation of c-Src activity by Cbp and lipid rafts, two cell lines were used, as follows: Csk-deficient mouse embryonic fibroblasts (MEFs) harboring a doxycycline (Dox)-inducible c-Src expression system (Csk^−/−^ MEF/pBKT2-c-Src), and c-Src-expressing Csk^−/−^ MEFs harboring a Dox-inducible Cbp expression system (Csk^−/−^ MEF/c-Src/pBKT2-Cbp) [30]. Dox-induced c-Src expression can induce cell transformation, and the c-Src-induced transformation is efficiently suppressed by Dox-induced Cbp expression as observed previously [30].

**Figure 2:**
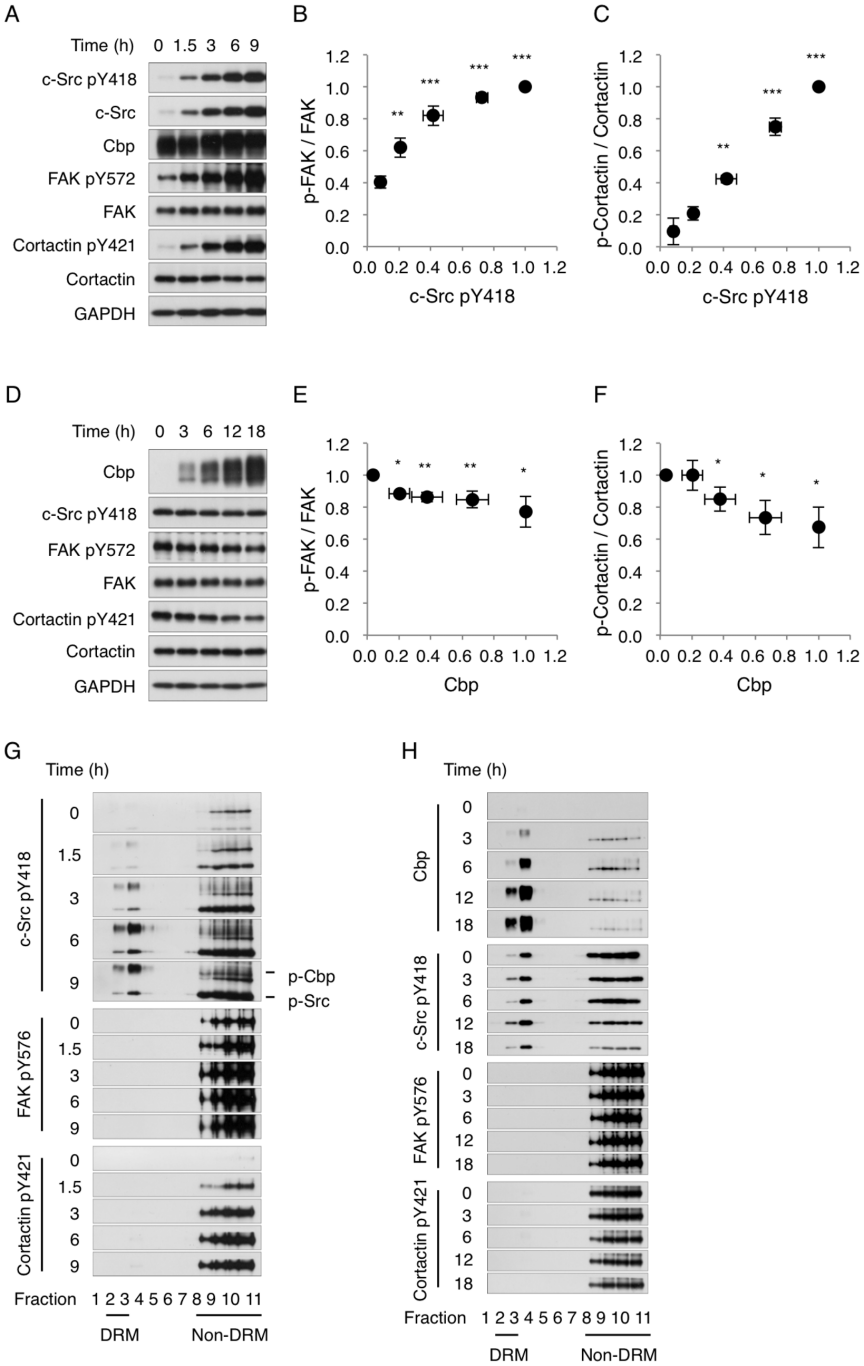
Phosphorylation status of FAK and cortactin following expression of c-Src and Cbp. (A and D) Csk^−/−^ MEFs harboring pBKT2-c-Src (Csk^−/−^ MEF/p-BKT2-c-Src cells, A) and c-Src-expressing Csk^−/−^ MEFs harboring pBKT2-Cbp (Csk^−/−^ MEF/c-Src/p-BKT2-Cbp cells, D) were incubated with or without 1 μg/ml Dox for the indicated time periods. Cell lysates from these cells were subjected to immunoblot analysis with the indicated antibodies. (B, C, E, and F) Relative intensity of phosphorylated FAK and cortactin as a function of c-Src and Cbp. The band intensities of c-Src pY418 and Cbp were normalized to the GAPDH band intensities, and then the relative intensities were calculated by dividing the band intensities of 9 h treated Csk^−/−^ MEF/p-BKT2-c-Src cells (B and C) and 18 h treated Csk^−/−^ MEF/c-Src/p-BKT2-Cbp cells (E and F). The band intensities of phosphorylated FAK and cortactin were normalized as p-FAK/FAK and p-cortactin/cortactin, respectively. The relative intensities were calculated in the same way as employed for the c-Src and Cbp intensity calculation. Means ± standard deviations (SD) were obtained from three independent experiments. (G and H) Csk^−/−^ MEF/p-BKT2-c-Src cells (G) and Csk^−/−^ MEF/c-Src/p-BKT2-Cbp cells (H) were incubated with or without 1 μg/ml Dox for the indicated time periods. DRM and non-DRM fractions of treated cells were separated on a sucrose density gradient. Aliquots of the fractions were immunoblotted with the indicated antibodies.

The expression and phosphorylation levels of c-Src were first determined. Analysis of whole-cell lysates showed that c-Src protein expression and phosphorylation at tyrosine 418 (pY418) were induced by Dox treatment in a time-dependent manner (Figure 2A). During this period, Cbp levels were unchanged, although there were band shifts due to phosphorylation by c-Src. The raft localization of activated c-Src was next assessed by separating detergent-resistant membrane (DRM) (lipid rafts) and non-DRM fractions (non-raft membranes). The DRM-separation assay showed that activated c-Src was predominantly localized to non-DRM fractions (Figure 2G), where FAK and cortactin were also localized [25], [31]. Following c-Src induction, phosphorylation of FAK at tyrosine 576 (pY576), which enhances its activity [16], was dramatically elevated (Figure 2A) in non-DRM fractions (Figure 2G). The relative intensity of FAK phosphorylation (p-FAK/FAK) increased as c-Src levels increased (Figure 2B). Likewise, c-Src induction increased the phosphorylation of cortactin at tyrosine 421 (pY421) (Figure 2A and 2C) in non-DRM fractions (Figure 2G). These observations demonstrate that c-Src activation induces concurrent phosphorylation of its substrates, FAK and cortactin, in non-raft membranes, a process associated with cell transformation [30].

In the Cbp-inducible cells, expression of Cbp was also induced by Dox treatment in a time-dependent manner (Figure 2D). However, c-Src activation (pY418) did not change during this time period (Figure 2D), indicating that Cbp overexpression does not inhibit total c-Src kinase activity. The DRM-separation assay showed that Cbp is exclusively distributed to DRM fractions, and that the levels of activated c-Src in non-DRM fractions decreased as Cbp levels increased (Figure 2H). This indicates that activated c-Src is sequestered into lipid rafts by Cbp overexpression. Consistent with the reduction of c-Src in non-DRM fractions, phosphorylation of FAK (p-FAK/FAK) and cortactin (p-cortactin/cortactin) was decreased in a Cbp-dependent manner (Figure 2D-2F and 2H). These finding support our previous observation that Cbp serves as a suppressor of activated c-Src by sequestering it into lipid rafts [25], [26], [31].

### Mathematical modeling

**Figure 3:**
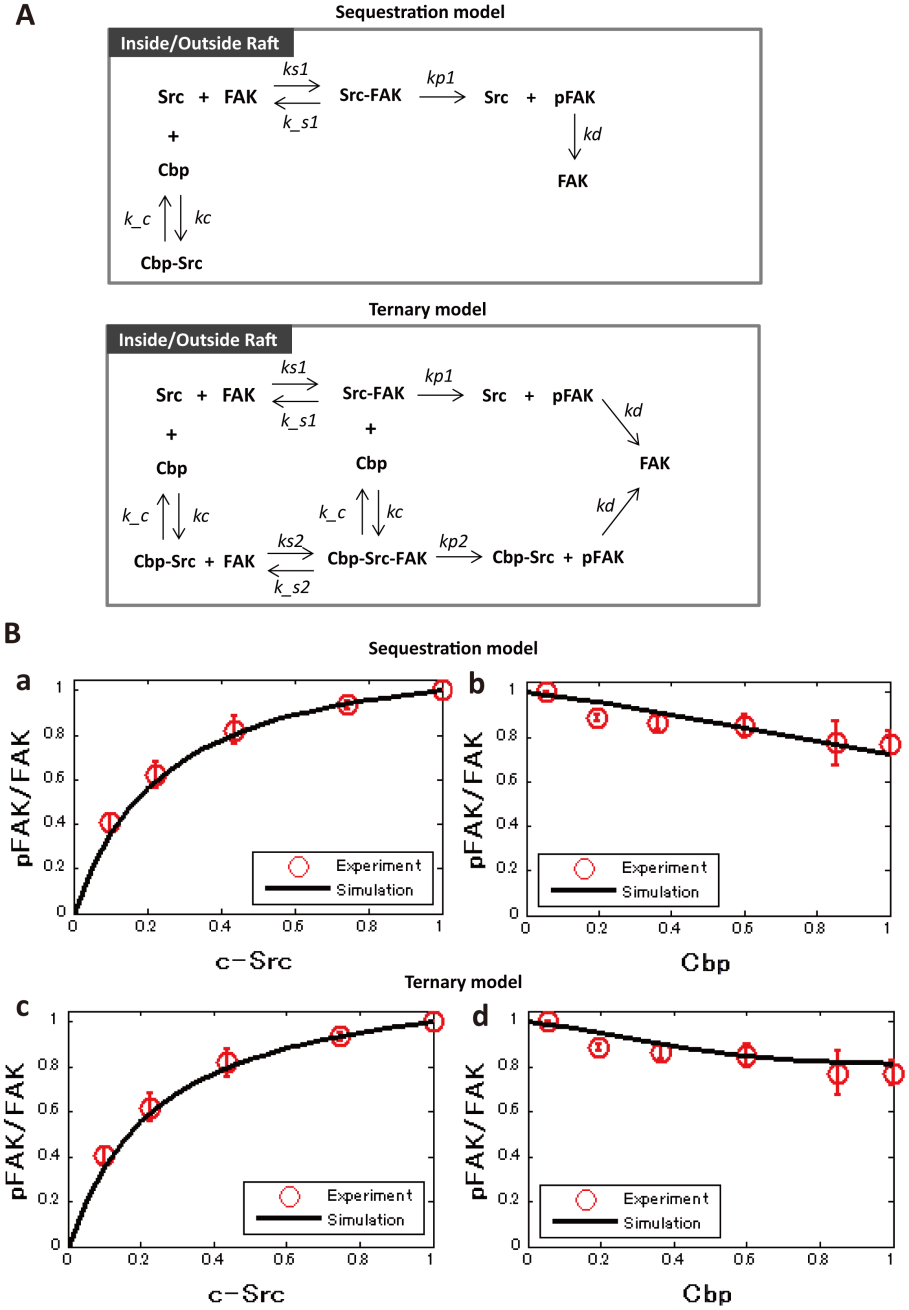
Schematic representation of mathematical models. (**A**) Two possible mechanisms are proposed based on the literature. c-Src binds to Cbp through its SH2 domain and forms a complex, Cbp-Src. c-Src also binds to FAK through its SH3 domain and phosphorylates it by a Michaelis-Menten mechanism. In the sequestration model, Cbp inhibits c-Src activity in a competitive way by sequestering c-Src. The ternary model was developed by adding the ternary complex consisting of Cbp, c-Src, and FAK to the sequestration model. Reaction schemes are assumed to be similar in raft and non-raft compartments. (B) Simulated phosphorylation curve of FAK. Simulated data are shown in black lines. Experimental data points depicted in red circles with bars show the mean ± SD (n = 3). (a) FAK phosphorylation ratio as a function of the total Src concentration for the sequestration model. (b) FAK phosphorylation ratio as a function of the total Cbp concentration for the sequestration model. (c) FAK phosphorylation ratio as a function of the total Src concentration for the ternary model. (d) FAK phosphorylation ratio as a function of the total Cbp concentration for the ternary model.

Based on the *in vitro* experimental data, mathematical models were next developed for the interactions between lipid rafts, c-Src, Cbp, and FAK. To construct possible models, we postulated that c-Src signaling is carried out through a series of tyrosine phosphorylation events in two cellular compartments, lipid rafts and non-raft membranes. All reactions were assumed to be the same in both lipid rafts and non-raft membranes. Two possible reaction schemes can be considered based on the literature. We previously showed that c-Src binds to Cbp through its SH2 domain and forms a complex, Cbp-Src [19], [25]. c-Src also binds to FAK through its SH2 domain [16], and possibly through its SH3 domain [32]. Therefore, by assuming that phosphorylation of FAK by c-Src occurs through a Michaelis-Menten-type reaction and that its dephosphorylation is simply described by first-order kinetics, we developed a simple model, the sequestration model (Figure 3A). However, the possibility remains that the Cbp-Src complex is able to bind to Src substrates (SS) and form a ternary complex, Cbp-Src-FAK, to phosphorylate FAK, which led us to develop an alternate model, the ternary model (Figure 3A). We then considered these two models to determine which one best reflects the system. The sequestration model consists of five biochemical reactions with three elementary species: activated c-Src, Cbp, and FAK, in two compartments. On the other hand, the ternary model consists of seven biochemical reactions with three elementary species: activated c-Src, Cbp, and FAK, in two compartments. In these two models, all biochemical reactions are modeled by a system of ordinary differential equations (ODEs). A schematic representation of the models is shown in Figure 3A. The full sets of equations are shown in Materials and Methods. The parameters are assigned as ***ks1***: Src and FAK binding constant, ***k_s1***: Src and FAK dissociation constant, ***ks2***: Cbp-Src and FAK binding constant, ***k_s2***: Cbp-Src and FAK dissociation constant, ***kc***: Cbp and Src binding rate constant, ***k_c***: Cbp and Src dissociation rate constant, ***kp1***: FAK phosphorylation rate constant through the Src-FAK complex, ***kp2***: FAK phosphorylation rate constant through Cbp-Src-FAK complex, and ***kd***: FAK dephosphorylation rate constant.

Shuttling behavior into and out of lipid rafts is described by first order kinetics, according to a previous report [33]. Thus, molecular localization in lipid rafts and non-raft membranes can be described by adjusting the rate constants. The experimental data show that Cbp is predominantly located to lipid rafts independently of c-Src-expression levels (Figure 2G), and that when Cbp expression is induced, c-Src is relocated to lipid rafts (Figure 2H). These results indicate that the Cbp-Src complex is distributed to lipid rafts. In contrast, FAK is mostly located to non-raft membranes (Figure 2G) [25], [31], enabling us to assume that the Src-FAK complex is formed in non-raft membranes and that the Cbp-Src-FAK complex can also be located in non-raft membranes. The parameters assigned are ***k_cin_***: import rate (into raft fractions) for Cbp and Cbp-Src, ***k_cout_***: export rate (from raft fractions) for Cbp and Cbp-Src, ***k_sin_***: import rate for Src, ***k_sout_***: export rate for Src, ***k_ssin_***: import rate for Src-FAK and Cbp-Src-FAK, and ***k_ssout_***: export rate for Src-FAK and Cbp-Src-FAK.

### Computational analysis revealed that only the ternary model includes the raft-volume dependence on FAK phosphorylation

**Figure 4:**
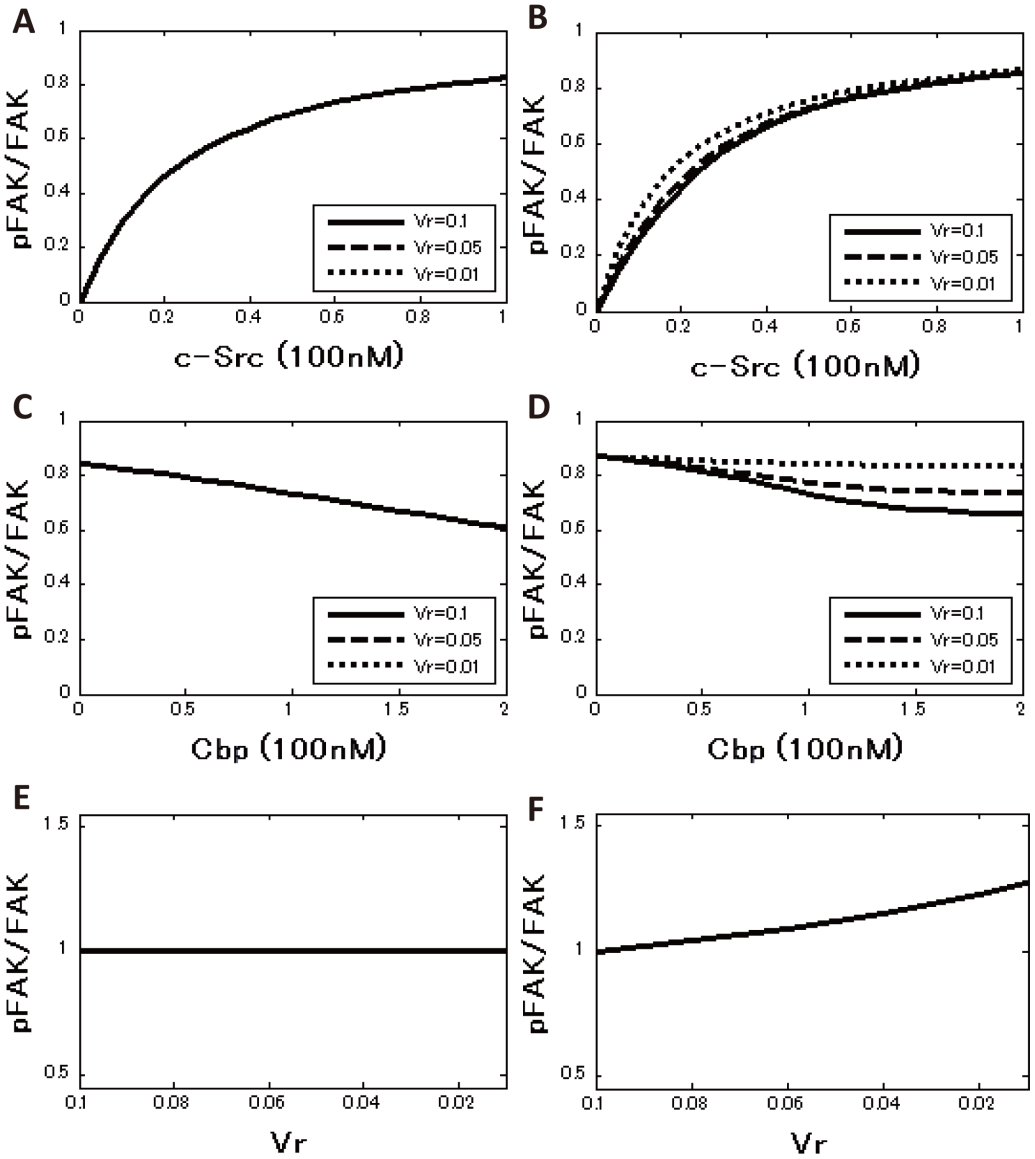
Simulated phosphorylation curve for the sequestration model and the ternary model. (A and B) FAK phosphorylation ratio as a function of the total c-Src concentration for three different raft-volume ratios in the sequestration model (A) and the ternary model (B). The black line denotes the raft-volume ratio ***Vr*** = 0.1 (control), the dashed line denotes ***Vr*** = 0.05, and the pointed line denotes ***Vr*** = 0.01. (C and D) FAK phosphorylation ratio as a function of the total Cbp concentration for three different raft-volume ratios in the sequestration model (C) and the ternary model (D). (E and F) The fold change in FAK phosphorylation level with respect to the raft-volume ratio ***Vr***. The values are normalized by ***Vr*** = 0.1.

Parameters were searched by random sampling (Text S1 and S2, Figure S1, Table S1), and the FAK phosphorylation curve obtained using the estimated parameter values was superimposed on the experimental data (Figure 3B and S2, Table S2 and S3). The properties of the steady-state solution of the two models were then compared. The dependence of raft volume on c-Src function was first examined by modulating ***Vr***, which represents the ratio of the raft volume to the total membrane volume, beginning with ***Vr*** = 0.1. In the sequestration model, decreasing ***Vr*** did not affect the phosphorylation level of FAK at any concentration of c-Src (Figure 4A), while in the ternary model, as the ***Vr*** decreased, the phosphorylation of FAK increased at all concentrations of c-Src (Figure 4B). Even when c-Src activity was analyzed as a function of the Cbp level, a decrease in ***Vr*** had no effect on the phosphorylation of FAK in the sequestration model (Figure 4C and 4E), while a decrease in ***Vr*** attenuated the inhibitory activity of Cbp on the phosphorylation of FAK in the ternary model (Figure 4D and 4F). Interestingly, when ***Vr*** was decreased to 0.01 (a condition where a large amount of Cbp is distributed to non-raft fractions) Cbp loses its inhibitory activity on c-Src function, irrespective of Cbp concentration (Figure 4D). We further investigated whether these properties depended on the initial choice of raft-volume ratio, ***Vr***. The raft-volume ratio was therefore set to ***Vr*** = 0.05, and the parameters were re-estimated by random sampling, as was done previously. The results showed that the raft-volume dependence is independent of the initial choice of ***Vr*** (Figure S3). These results support our proposal that raft localization of Cbp is required to exert an inhibitory effect on c-Src function. Since Cbp is exclusively localized to lipid rafts under normal conditions, the model reflects our *in vitro* observations and underscores the importance of lipid rafts in the negative regulation of c-Src.

### Experimental validation of the raft volume dependence on FAK phosphorylation

**Figure 5:**
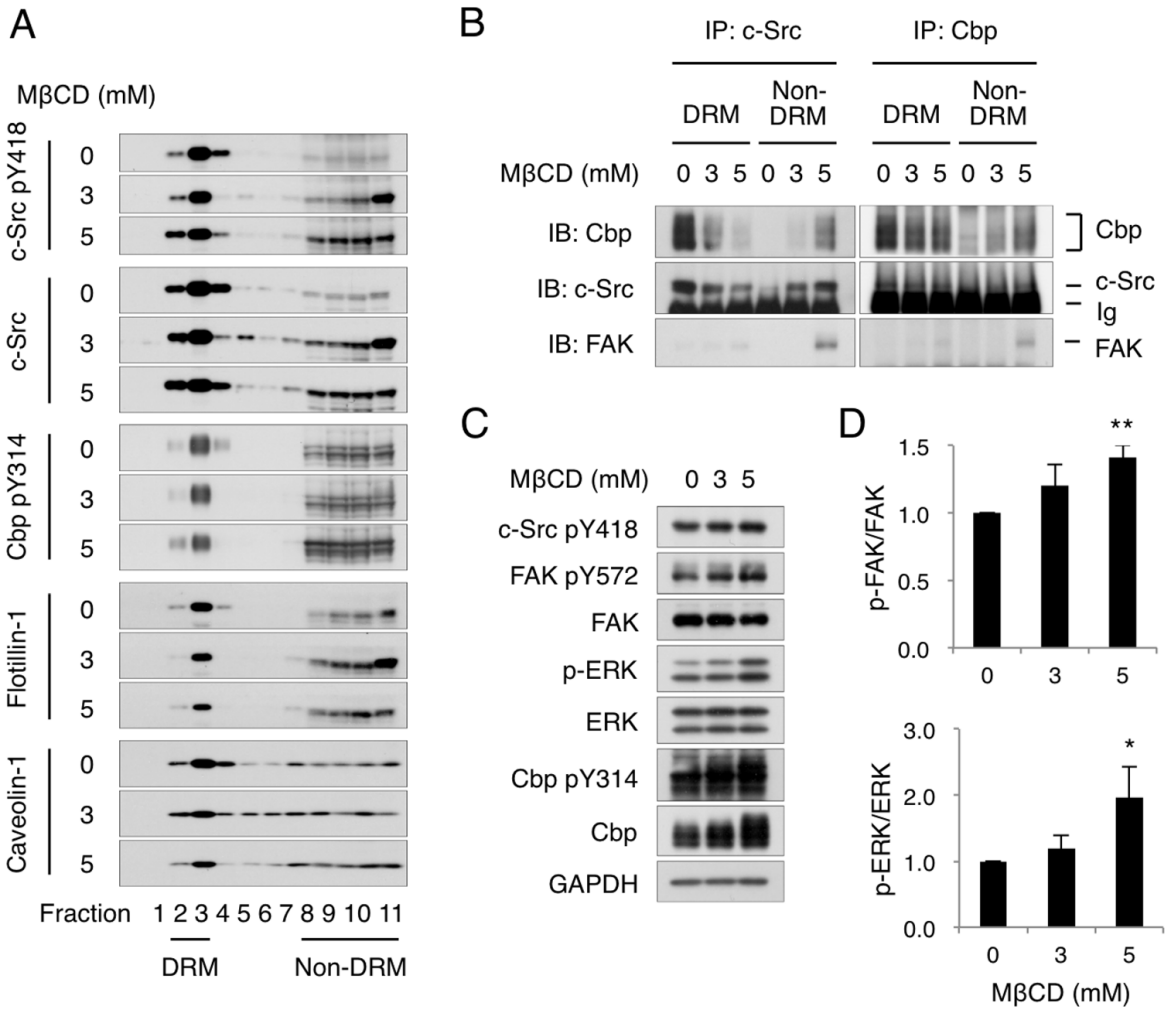
Experimental validation and prediction for the ternary model. (A) Csk^−/−^ MEFs were incubated with MβCD at the indicated concentrations for 1 h. DRM and non-DRM fractions of treated cells were separated on sucrose density gradient. Aliquots of the fractions were immunoblotted with the indicated antibodies. (B) DRM and non-DRM fractions of the MβCD-treated Csk^−/−^ MEFs were subjected to immunoprecipitation (IP) with anti-Src or anti-Cbp antibodies, followed by immunoblotting with the indicated antibodies. Ig, immunoglobulin heavy chain. (C) Total cell lysates from the MβCD-treated Csk^−/−^ MEFs were subjected to immunoblot analysis with the indicated antibodies. (D) The phosphorylation status of FAK (p-FAK/FAK) and ERK (p-ERK/ERK) was obtained from the band intensities of panel C and calculated by defining the value for non-treated controls as 1.0. The relative specific activities were calculated by defining the value for non-treated controls as 1.0. Means ± SD were obtained from three independent experiments. ^*^ *P*<0.05, ^**^ *P*<0.01, Student's *t*-test.

To determine which model is more appropriate, the raft-volume dependence on FAK phosphorylation was examined in *in vitro* experiments. Based on the ternary model, when Cbp is excluded from lipid rafts, the active Cbp-Src complex can efficiently phosphorylate non-raft substrates. To validate this proposal, the effect of forced exclusion of Cbp from lipid rafts on c-Src function was examined. For this, lipid rafts were disrupted by depleting cholesterol with methyl-β-cyclodextrin (MβCD) in Csk-deficient cells. Treatment with MβCD reduced cholesterol levels (data not shown) and induced the relocation of the raft marker proteins flotillin-1 and caveolin-1 from DRM fractions to non-DRM fractions (Figure 5A), indicating that lipid rafts were successfully disrupted. Under these conditions, active c-Src and phosphorylated Cbp were relocated from DRM fractions to non-DRM fractions (Figure 5A). An immunoprecipitation assay revealed that c-Src and Cbp interacted with each other, and that the Cbp-Src complex was relocated to non-DRM fractions in a manner dependent on MβCD concentration (Figure 5B). Furthermore, association of FAK with this complex was detected only in non-DRM fractions (Figure 5B). Consistent with a previous report [26], FAK phosphorylation was significantly enhanced by MβCD treatment (Figure 5C and 5D). Phosphorylation of ERK, a downstream component of FAK signaling, was also significantly increased, confirming the activation of the FAK signaling pathway. These observations demonstrate that the ternary complex, Cbp-Src-FAK, is indeed formed in non-raft membranes and that the phosphorylation of FAK increases when raft volume is reduced. These *in vitro* experimental results are in agreement with the assumptions and simulation results of the ternary model (Figure 4B, 4D and 4F).

### Computational analysis revealed that enhancement of the association of c-Src with FAK by Cbp is required for the positive regulatory role of Cbp

**Figure 6:**
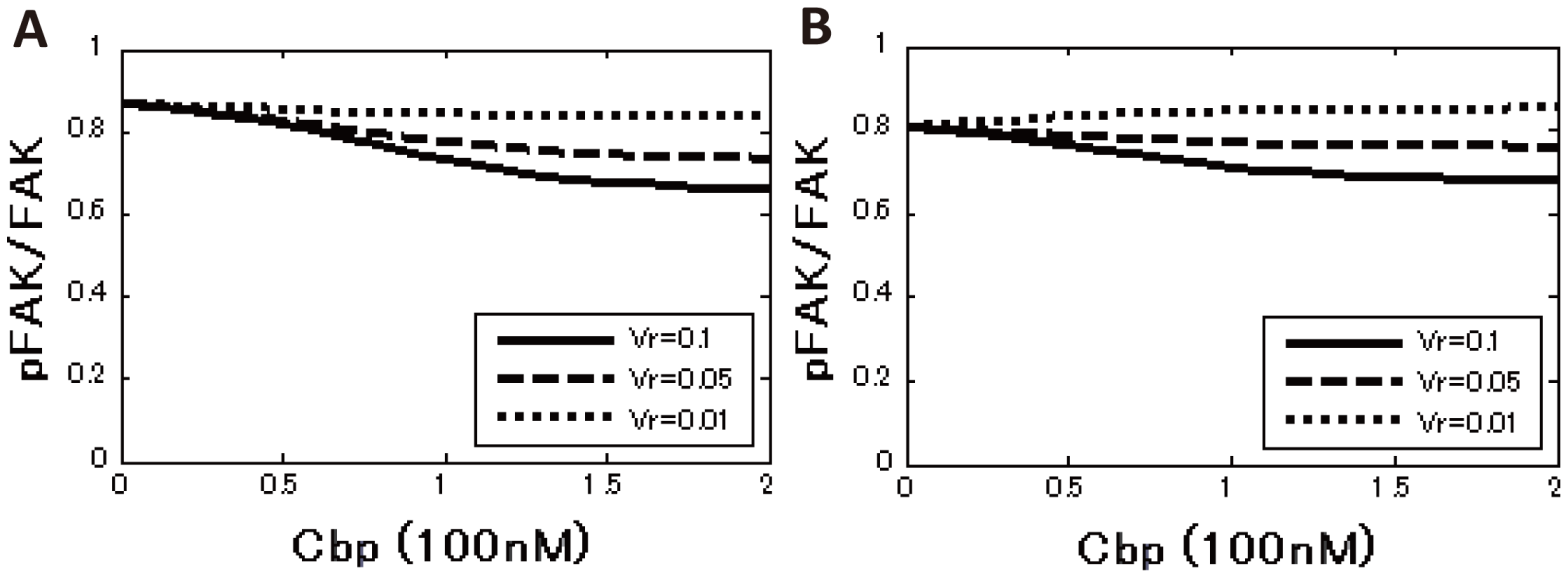
Simulated phosphorylation curve for the effects of Cbp on c-Src function. (A) FAK phosphorylation ratio as a function of the total Cbp concentration. The phosphorylation rate constant is set as kp2 = 2*kp1 (***α*** = 2). (B) FAK phosphorylation ratio as a function of the total Cbp concentration. The Src-FAK binding rate constant is set as ks2 = 2*ks1 (***β*** = 2).

To evaluate the potential positive role of Cbp in c-Src regulation, computational analysis was again performed by adopting the ternary model. We previously found that phosphorylated Cbp binds Csk tyrosine kinase at its SH2 domain and enhances its catalytic activity [23]. Thus, the contribution of c-Src activation by Cbp was input into the ternary model. The rate constants were first set as ***ks2*** = ***ks1***, ***k_s2*** = ***k_s1***, and ***kp2*** = ***α*** *•* ***kp1*** (***α = 2***), where ***α*** is defined as the activating potential of the rate of FAK phosphorylation, and then random parameter sets were re-sampled and the parameter values were re-estimated. As in the previous result (Figure 4D), the phosphorylation of FAK decreased as Cbp increased, while Cbp lost its inhibitory activity on c-Src function irrespective of Cbp concentration when ***Vr*** was decreased to 0.01 (Figure 6A). Setting the activating potential ***α*** = 10 resulted in no significant difference from the ***α = 2*** results (Figure S4A), indicating that sequestration of c-Src into lipid rafts by binding to Cbp can suppress c-Src function even though c-Src catalytic activity is enhanced at least 10-fold. The rate constants were next set as ***ks2*** = ***β*** *•* ***ks1*** (***β*** **= 2**), ***k_s2*** = ***k_s1***, and ***kp2*** = ***kp1***, where ***β*** is defined as the activating potential of the Src/FAK binding rate. When ***Vr*** = 0.1, the phosphorylation of FAK decreased as Cbp increased. However, when ***Vr*** was set to 0.01, the phosphorylation of FAK was slightly elevated relative to the levels observed before Cbp induction (Figure 6B). When ***α*** and ***β*** were set to 2 to investigate the synergy of the activating potentials ***α*** and ***β***, enhancement of FAK phosphorylation was also observed, but no significant difference was observed compared to the case when ***α*** = 1 and ***β*** = 2 (Figure S4B). The effect of the initial choice of raft-volume ratio, ***Vr***, on these properties was next investigated. The results showed that the raft-volume dependence was independent of the initial choice of ***Vr*** (Figure S5), suggesting that enhancement of affinity for substrates (***ks2***) can contribute to the positive effect of Cbp on c-Src function. Therefore, these observations imply that if Cbp directly induces the association of c-Src with FAK, Cbp can exert a positive effect on c-Src function when the Cbp-Src complex is delivered to non-raft membranes, where it can encounter substrates.

## Discussion

This study addressed molecular mechanisms of the spatial regulation of c-Src by combining mathematical modeling and *in vitro* experimental approaches. Based on initial experimental data, two mathematical models, the sequestration model and the ternary model, were developed, and their properties were investigated by random sampling approaches. Both models support our proposal that raft localization of Cbp is crucial for suppression of c-Src function, but the ternary model predicts the dependence of c-Src function on the lipid-raft volume. Raft disruption experiments using MβCD showed that c-Src function depends on raft volume and that a ternary complex consisting of Cbp, c-Src and FAK is formed in non-raft fractions, indicating that the ternary model best describes the system. To address the potential positive role of Cbp, computational analysis was also performed by setting the ternary model to include the effect of Cbp binding on c-Src catalytic activity. Analyses using an increased binding constant for the c-Src and FAK complex suggest that if Cbp is excluded from lipid rafts, it can positively regulate c-Src activity. This prediction was supported by the previous observation that disruption of lipid rafts in Csk-deficient cells induced c-Src-mediated cell transformation [26], although detailed experimental analysis will be necessary to elucidate the mode of c-Src activation. These findings not only underscore the crucial role of lipid rafts in the Cbp-mediated negative regulation of c-Src transforming activity, but may also explain the positive regulation of c-Src by Cbp under certain conditions where lipid rafts are perturbed.

To analyze the spatial regulation of the transforming activity of c-Src, Csk-deficient cells were used as a model system. Cbp serves as an initial substrate of c-Src, and phosphorylated Cbp recruits cytosolic Csk to lipid rafts to allow close contact with c-Src [19]. Since this negative feedback mechanism occurs constitutively, it has been difficult to analyze the initial events of c-Src activation in normal fibroblasts. In some cancer cells, however, a substantial population of c-Src is present as an active form even though Csk is expressed [25]. Therefore, to dissect the initial molecular events following c-Src activation, and to mimic the activity status of c-Src in cancer cells, we used Csk-deficient cells. This system was successfully used to detect the direct association between activated c-Src and Cbp, and characterize the role of raft-anchored Cbp as a negative regulator of c-Src-mediated transformation. This tumor-suppressive function of Cbp has been verified in v-Src-transformed cells and some human cancer cells, both of which express Csk [26]. We previously found in this system that disruption of lipid rafts was sufficient to induce robust cell transformation [26]. Analysis of this phenomenon showed that liberation of c-Src from lipid rafts allows c-Src to make contact with non-raft substrates, such as FAK, which triggers cell transformation. To validate this mechanism and gain new insights into the mechanisms regulating cell transformation, we conducted a simulation study using a c-Src- or Cbp-inducible system as an experimental model [30]. However, since Csk is ubiquitously expressed even in cancer cells [34], Csk function will be incorporated into these models in follow-up studies, particularly those investigating regulatory mechanisms for stimulus-dependent c-Src function.

c-Src activity is regulated by intramolecular domain interaction [6]. When the negative regulatory tyrosine in the C-terminus is phosphorylated, its own SH2 domain interacts with this site to adopt a closed conformation that cannot access substrates. As phosphorylated Cbp directly interacts with c-Src SH2 domain [19], [26], it is likely that Cbp can induce a conformation change in c-Src, resulting in an increase in its catalytic activity as well as its affinity for substrates. Computational analysis revealed that if Cbp activates the association of c-Src with FAK, Cbp could exert a positive effect on c-Src function. Since c-Src interacts with FAK through its SH2 and/or SH3 domain [32], [35], it is possible that the binding of Cbp to the c-Src SH2 domain may promote the interaction of FAK with the c-Src SH3 domain. Although the complete mechanisms of this interaction must await future study, the association of FAK with the Cbp-Src complex would further enhance the mutual activation between c-Src and FAK, promoting cell transformation, as observed in the previous study [25].

Scaffold proteins play key roles in regulating signal transduction [36]–[39], physically assembling pathway components and sequestering them into subcellular compartments. By binding and spatiotemporally organizing pathway components, scaffold proteins promote cell signaling along a particular pathway. Our analysis showed that Cbp plays a scaffolding role in c-Src signaling by sequestering c-Src and its substrates. Because c-Src serves as a signaling hub in response to diverse extracellular stimuli, Cbp may regulate the multiple functions of c-Src by spatiotemporally controlling c-Src activity. Therefore, it would be interesting to investigate how Cbp is involved in controlling stimulus-dependent c-Src signaling.

The SFKs Fyn and Lyn also interact with Cbp through their SH2 and SH3 domains [28], [40], [41], suggesting that Cbp is involved in the regulation of all SFKs. The function of Fyn and Lyn in early events in B cell antigen-receptor (BCR) signaling was computer-simulated based on a model incorporating site-specific protein-protein interactions among BCR, Lyn, Fyn, Syk, Csk, and Cbp/PAG1 [42]. By incorporating the Cbp/PAG1-Csk complex as a negative regulator of SFKs, the analysis successfully reconstituted the function of Fyn and Lyn in BCR signaling, although the subcellular localization of signaling molecules was not considered in that study. On the other hand, it has been reported in a lymphoma cell line that the Cbp-Lyn complex phosphorylates signal transducer and activator of transcription 3 (STAT3) in lipid rafts, resulting in the efficient activation of transforming signals [29]. This finding demonstrates that Cbp can positively regulate SFKs when SFK substrates are present in lipid rafts. Because SFKs other than c-Src and Blk have a high affinity for lipid rafts and their raft localization is independent of Cbp [26], it is likely that intrinsic substrates for these SFKs are located in lipid rafts. Therefore, Cbp may function as a positive regulator specifically for these raft resident SFKs. By contrast, raft localization of c-Src is highly dependent on Cbp [26] and c-Src substrates, e.g., FAK and cortactin, being distributed in non-raft membranes. Due to these unique features of c-Src, Cbp can specifically act as a suppressor of c-Src by sequestering it into lipid rafts to limit its access to its substrates. However, as predicted by computational analysis, it is also possible that Cbp positively regulates c-Src when c-Src substrates are accumulated in lipid rafts or when the Cbp-Src complex is delivered to non-raft membranes under certain conditions where lipid rafts are perturbed. Cell transformation induced by MβCD in Csk-deficient cells might be mediated by Cbp acting in this fashion in non-raft membranes.

The ternary model showed that c-Src function is dependent on the lipid-raft volume. To validate this prediction, lipid rafts were disrupted by artificial treatment with MβCD [26]. However, we found that exclusion of Cbp from lipid rafts is an early event in cell transformation that occurs because of alterations in lipid-raft cholesterol and sphingolipid content [43]. This observation suggests that Cbp has the potential to serve as an activator of c-Src during cancer development when lipid metabolism is dysregulated. It is also possible that other pathological conditions that disturb lipid metabolism, such as aging, starvation, diabetes mellitus, or metabolic disorders, would allow Cbp to activate c-Src in non-raft membranes. Another possible mechanism is depalmitoylation or fatty acid remodeling of Cbp [44]. Because a mutant Cbp lacking a palmitoylation site loses its raft localization [25], perturbation of palmitoylation metabolism under some conditions may also contribute to the positive effect of Cbp on c-Src function. By contrast, we also observed that Cbp is downregulated in fully transformed cells [25] and that Cbp expression is silenced in cancer cells potentially through an epigenetic mechanism [45]. Re-expression of Cbp in c-Src transformed cells and some cancer cells successfully suppressed malignant progression of these cells by sequestering active c-Src into lipid rafts [25]. These findings clearly demonstrate that Cbp serves as a suppressor of Src-mediated tumor progression when it is localized to lipid rafts. Overall, it is possible that the function of Cbp is dependent on its localization, exhibiting tumor-suppressive activity in lipid rafts and tumor-promoting activity in non-raft membranes. The ternary model clearly predicted these reciprocal functions of Cbp. Therefore, given that appropriate parameters in particular cancer cells are available, the mathematical models constructed in this study would be applicable to predict the behavior of Src-activated cancer cells [46]–[48].

In conclusion, our combination analysis unveiled opposing roles for Cbp in the regulation of transforming c-Src. Using computational models, we were able to identify a positive regulatory role for Cbp localized to non-raft membranes, an these models highlighted the importance of the subcellular localization of c-Src substrates. The modeling study also produced new interesting questions that must be addressed, plus the need for the molecular basis for the strict barrier function of lipid rafts against non-raft membranes to be elucidated, the physiological relevance of raft perturbation to c-Src activation to be widely investigated, and the identification of lipid-raft-residing c-Src substrates which would shed new light on the normal function of lipid-raft-anchored c-Src. The establishment of a mathematical model that fully represents the c-Src regulatory system would be useful for *in silico* screening of therapeutic targets in human cancers in which c-Src is upregulated.

## Materials and Methods

### Cell and cell culture

Csk-deficient (*Csk^−/−^*) MEFs were a kind gift from Dr. A Imamoto [49]. Csk^−/−^ MEFs transfected with pBKT2-c-Src (Csk^−/−^/p-BKT2-c-Src) and *Csk^−/−^* MEFs transfected with both c-Src and pBKT2-Cbp (Csk^−/−^/c-Src/p-BKT2-Cbp) were kindly donated by Dr. F Imamoto [30]. Cells were cultured in Dulbecco's modified Eagle's medium (DMEM) supplemented with 10% fetal bovine serum (FBS). For induction of c-Src and Cbp expression, cells were incubated with 1 mg/ml doxycycline (Dox, Sigma).

### Western blot analysis

Cells were washed with PBS and lysed with n-octyl-β-D-glucoside (ODG) buffer [20 mM Tris-HCl (pH 7.4), 150 mM sodium chloride, 1 mM EDTA, 1 mM sodium orthovanadate, 20 mM sodium fluoride, 1% Nonidet P-40, 5% glycerol, 2% ODG, and a protease inhibitor cocktail], and immunoblotting was carried out as described previously [26]. The following primary antibodies were used: anti-Src (Ab-1; Calbiochem), anti-Src pY418 (Invitrogen), anti-phosphotyrosine (4G10; Millipore), anti-cortactin (Sigma), anti-cortactin pY421 (Sigma), anti-FAK (Santa Cruz), anti-FAK pY576 (Cell Signaling), anti-ERK pT202/Y204 (Cell Signaling), anti-ERK (Cell Signaling), anti-GAPDH (Sigma), anti-Flottilin-1 (BD) and anti-Caveolin-1 (BD). Anti-Cbp and anti-Cbp pY314 were prepared as described previously [19]. GAPDH was used as a loading control. Horseradish peroxidase (HRP)–conjugated anti-mouse or anti-rabbit IgG (Zymed) was used as the secondary antibody. All blots were visualized and quantitated using a LAS-4000 luminescent image analyzer (GE Healthcare).

### DRM fractionation

Fractionation of membrane compartments on a sucrose gradient was performed as described previously [26], . Cells were washed with PBS, lysed with homogenization buffer [50 mM Tris-HCl (pH 7.4), 150 mM sodium chloride, 1 mM EDTA, 1 mM sodium orthovanadate, 20 mM sodium fluoride, 0.25% Triton X-100 and a protease inhibitor cocktail] and separated on a discontinuous sucrose gradient (5-35-40%) by ultracentrifugation at 150,000×g for 12 h at 4°C using an Optima L-100XP with a SW55Ti (Beckman Coulter). Eleven fractions were collected from the top of the sucrose gradient.

### Immunoprecipitation

Fractions of membrane compartments were solubilized with 2% ODG and 1% Triton X-100 for 30 min at 4°C. The solubilized fractions were incubated with the indicated antibodies for 1 h at 4°C, and then further incubated with protein A-sepharose beads (GE healthcare) for 1 h at 4°C. The immunoprecipitates were washed with ODG buffer and analyzed by Western blotting.

### Kinetic modeling and numerical computation

The model is described by a system of ODEs. The details of the model are provided below. Numerical simulations of ODEs were implemented by MATLAB software (The Mathworks Inc.) using the ode23 function. In all simulations, the time evolution was performed up to the time *T* = 1,000 sec. Parameter estimation was performed by random sampling. The essence of the analysis is minimization of the target function, which was chosen as the residual sum of squares (RSS) between the experimental data and the simulation data,

**RSS = (Σ Src data+Σ Cbp data){Experimental data – Simulated data}^2^**,

where “Src data” and “Cbp data” show data sequences as the Src and Cbp concentrations change. For details, see Text S1 and S2, Table S1, S2 and S3.

### Ordinary differential equations

Here, we present a system of ODEs for the sequestration model and the ternary model. The differences between the sequestration model and the ternary model are the existence of a ternary complex, Cbp-Src-FAK, and its related kinetic reactions. In the sequestration model, the system of ordinary differential equations is composed of 12 variables. The equations are given by:
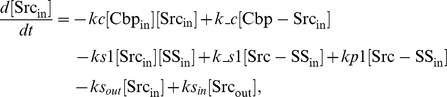


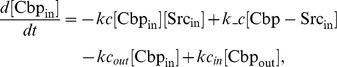





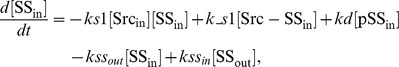


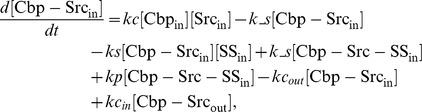


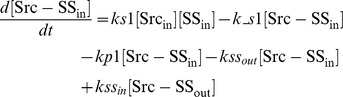


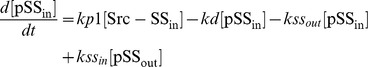


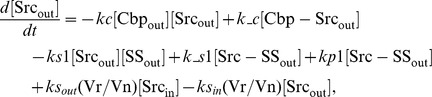


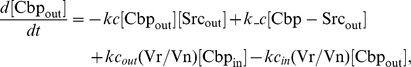


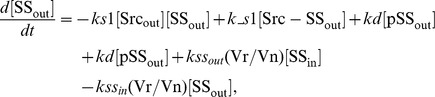


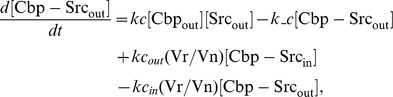


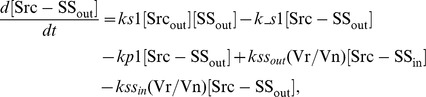


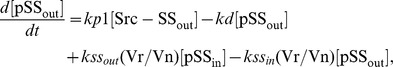



In the ternary model, the system of ordinary differential equations is composed of 14 variables. The equations are given by:
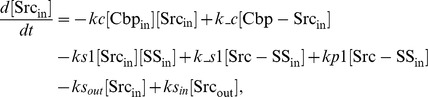


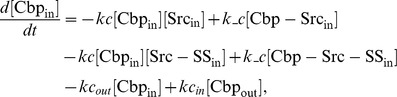


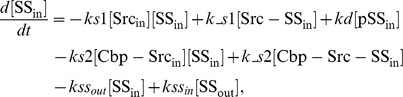


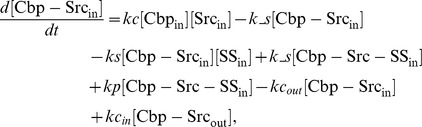


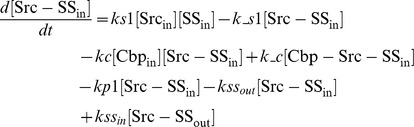


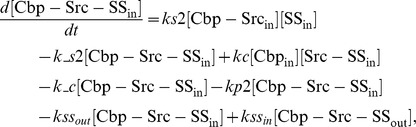


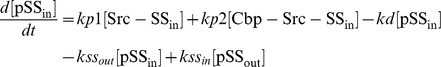


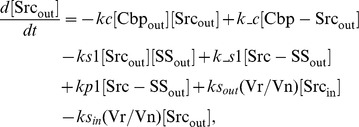


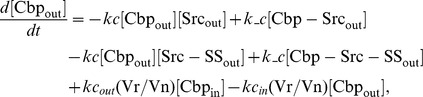


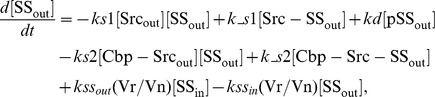


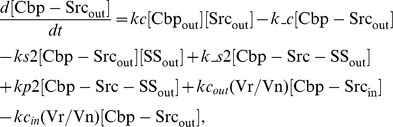


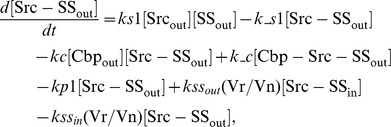


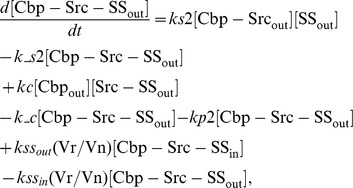


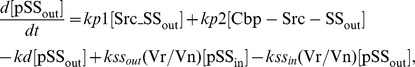



## Supporting Information

Figure S1 **Schematic representation of the subsystems.** (A) Michaelis-Menten-type reaction of c-Src mediated phosphorylation of SS. (B) c-Src and Cbp binding and dissociation reaction. (C) Import into and export from membrane microdomains.
(TIF)

Figure S2 **Results of random sampling for parameter estimation.** Two-dimensional scatter plots in the ***K_M_*** and ***K_dep_*** plane for the sequestration model (A) and the ternary model (B). Green dots indicate parameters satisfying RSS>0.03, red dots indicate parameters satisfying 0.015<RSS<0.03, and blue dots indicate parameters satisfying RSS<0.015.
(TIF)

Figure S3 **Simulated phosphorylation curve for the sequestration model and the ternary model with** ***Vr*** **= 0.05.** (A and B) FAK phosphorylation ratio as a function of the total Src concentration for three different raft volume ratios in the sequestration model (A) and the ternary model (B). The black line denotes the raft volume ratio ***Vr*** = 0.05 (control), the dashed line denotes ***Vr*** = 0.01, and the pointed line denotes ***Vr*** = 0.001. (C and D) FAK phosphorylation ratio as a function of the total Cbp concentration for three different raft volume ratios in the sequestration model (C) and the ternary model (D).
(TIF)

Figure S4 **Simulated phosphorylation curve for allosteric effects.** (A) FAK phosphorylation ratio as a function of the total Cbp concentration. The phosphorylation rate constant is set as ***kp2*** **=** 10*kp1 (***α*** = 10). (B) FAK phosphorylation ratio as a function of the total Cbp concentration. The phosphorylation rate constant is set as kp2 = 2*kp1 (***α*** = 2) and the Src-FAK binding rate constant is set as ks2 = 2*ks1 (***β*** = 2).
(TIF)

Figure S5 **Simulated phosphorylation curve for allosteric effects with** ***Vr*** **= 0.05.** (A) FAK phosphorylation ratio as a function of the total Cbp concentration. The phosphorylation rate constant is set as kp2 = 2*kp1 (***α*** = 2). (B) FAK phosphorylation ratio as a function of the total Cbp concentration. The Src-FAK binding rate constant is set as ks2 = 2*ks1 (***β*** = 2).
(TIF)

Table S1 **Search range for parameter estimation.**
(DOCX)

Table S2 **Parameter values for the sequestration model with** ***Vr*** **= 0.1.**
(DOCX)

Table S3 **Parameter values for the ternary model with** ***Vr*** **= 0.1.**
(DOCX)

Text S1 **Analysis of subsystems.**
(DOCX)

Text S2 **Random sampling for parameter searches.**
(DOCX)
