# Supplementary figures and images for: Roles of Raft-Anchored Adaptor Cbp/PAG1 in Spatial Regulation of c-Src Kinase
Source: PLoS One. 2014 Mar 27;9(3):e93470. doi: 10.1371/journal.pone.0093470 (PMC3968143; doi:10.1371/journal.pone.0093470)

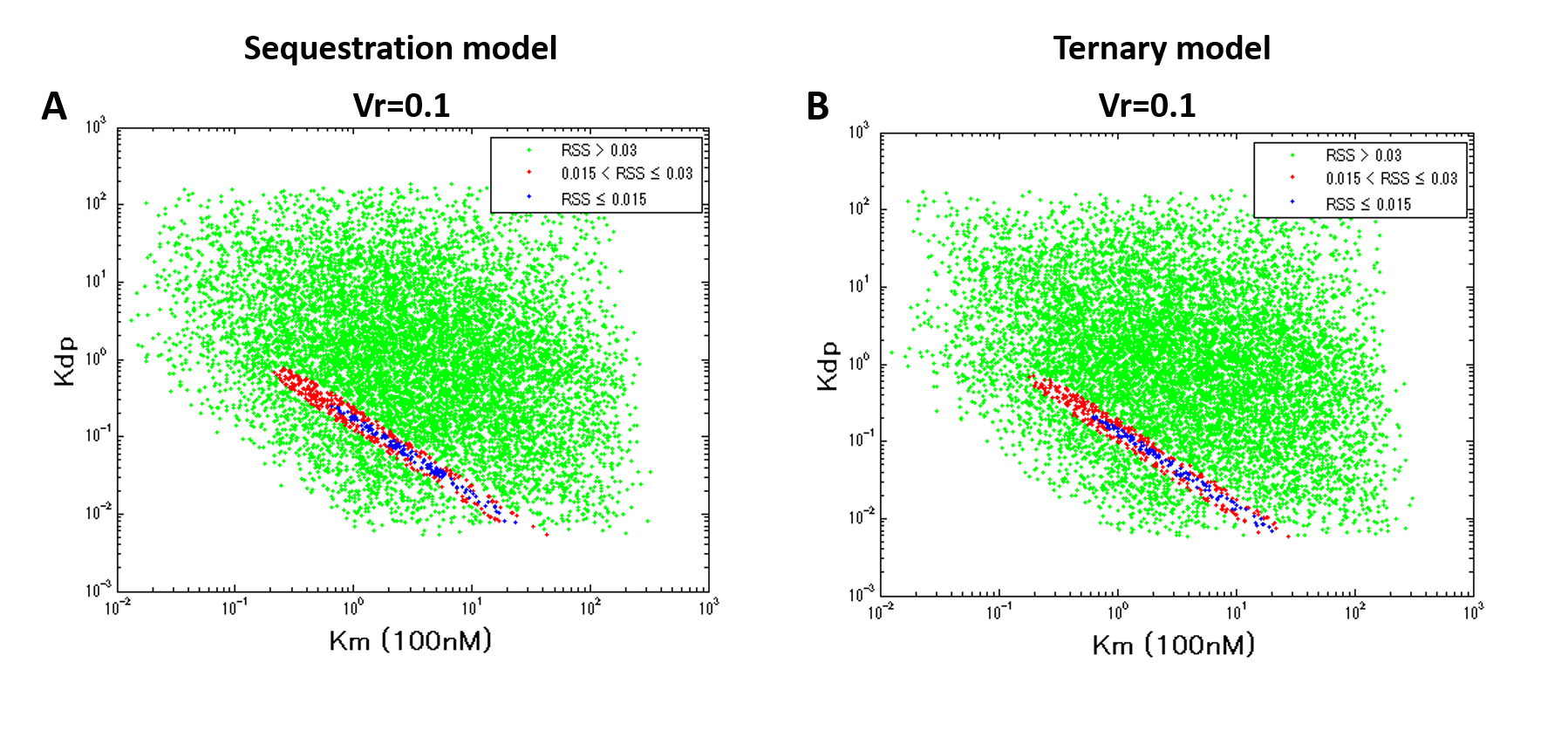

Supplement: Figure S2 — Results of random sampling for parameter estimation. Two-dimensional scatter plots in the KM and Kdep plane for the sequestration model (A) and the ternary model (B). Green dots indicate parameters satisfying RSS>0.03, red dots indicate parameters satisfying 0.015<RSS<0.03, and blue dots indicate parameters satisfying RSS<0.015. (TIF) [file pone.0093470.s002.tif]
