# Supplementary material for: Roles of Raft-Anchored Adaptor Cbp/PAG1 in Spatial Regulation of c-Src Kinase
Source: PLoS One. 2014 Mar 27;9(3):e93470. doi: 10.1371/journal.pone.0093470 (PMC3968143; doi:10.1371/journal.pone.0093470)
Supplement: Table S1 — Search range for parameter estimation. (DOCX) [file pone.0093470.s006.docx]

**Table S1. Search range for parameter estimation.**

| Symbol | Values | Units |
| --- | --- | --- |
| *ks1* | 0.05–10 | μM^-1^ s^-1^ |
| *k_s1* | 0.005–1 | s^-1^ |
| *ks2* | 0.05–10 | μM^-1^ s^-1^ |
| *k_s2* | 0.005–1 | s^-1^ |
| *kp1* | 0.005–1 | s^-1^ |
| *kp2* | 0.005–1 | s^-1^ |
| *kd* | 0.005–1 | s^-1^ |
