# Supplementary material for: Roles of Raft-Anchored Adaptor Cbp/PAG1 in Spatial Regulation of c-Src Kinase
Source: PLoS One. 2014 Mar 27;9(3):e93470. doi: 10.1371/journal.pone.0093470 (PMC3968143; doi:10.1371/journal.pone.0093470)
Supplement: Table S3 — Parameter values for the ternary model with Vr = 0.1. (DOCX) [file pone.0093470.s008.docx]

**Table S3. Parameter values for the ternary model with *Vr* = 0.1.**

| Symbol | Values | Units | Comments |
| --- | --- | --- | --- |
| *kc* | 10 | μM^-1^ s^-1^ | Fixed |
| *k_c* | 0.05 | s^-1^ | Fixed |
| *ks1* | 0.85 | μM^-1^ s^-1^ | Fitted |
| *k_s1* | 0.69 | s^-1^ | Fitted |
| *ks2* | 0.85 | μM^-1^ s^-1^ | Fitted |
| *k_s2* | 0.69 | s^-1^ | Fitted |
| *kp1* | 0.76 | s^-1^ | Fitted |
| *kp2* | 0.76 | s^-1^ | Fitted |
| *kd* | 0.006 | s^-1^ | Fitted |
| *kc_in_* | 0.36 | s^-1^ | Estimated from Figure 2 |
| *kc_out_* | 0.01 | s^-1^ | Estimated from Figure 2 |
| *ks_in_* | 0.1 | s^-1^ | Estimated from Figure 2 |
| *ks_out_* | 0.1 | s^-1^ | Estimated from Figure 2 |
| *kss_in_* | 0.01 | s^-1^ | Estimated from Figure 2 |
| *kss_out_* | 0.11 | s^-1^ | Estimated from Figure 2 |
| [FAK]_tot_ | 0.05 | μM | Fixed |
