# Supplementary material for: Roles of Raft-Anchored Adaptor Cbp/PAG1 in Spatial Regulation of c-Src Kinase
Source: PLoS One. 2014 Mar 27;9(3):e93470. doi: 10.1371/journal.pone.0093470 (PMC3968143; doi:10.1371/journal.pone.0093470)
Supplement: Text S1 — Analysis of subsystems. (DOCX) [file pone.0093470.s009.docx]

**Analysis of subsystems**

The full system can be divided into three parts: the phosphorylation and dephosphorylation of Src substrate (SS) (Figure S1A); the Cbp and Src binding and dissociation process (Figure S1B); and the import of c-Src into, and export of c-Src from, membrane microdomains (Figure S1C). Each process is described by the system of ordinary differential equations in the following forms:

$$\begin{matrix} \frac{d\text{[Src]}}{dt}=-ks\text{[Src][SS]}+k\text{\_}s\text{[Src-SS]}+kp\text{[Src-SS],} \\ \frac{d\text{[SS]}}{dt}=-ks\text{[Src][SS]}+k\text{\_}s\text{[Src-SS]}+kd\text{[pSS],} \\ \frac{d\text{[Src-SS]}}{dt}=ks\text{[Src][SS]}-k\text{\_}s\text{[Src-SS]}-kp\text{[Src-SS],} \\ \frac{d\text{[pSS]}}{dt}=kp\text{[Src-SS]}-kd\text{[pSS],} \end{matrix}$$

with the conservation laws $\text{[Src]}_{\text{tot}}=\text{[Src]}+\text{[Src-SS]}$, $\text{[SS]}_{\text{tot}}=\text{[SS]}+\text{[Src-SS]}+\text{[pSS]}$.

$$\begin{matrix} \frac{d\text{[Cbp]}}{dt}=-kc\text{[Cbp][Src]}+k\text{\_}c\text{[Cbp-Src],} \\ \frac{d\text{[Src]}}{dt}=-kc\text{[Cbp][Src]}+k\text{\_}c\text{[Cbp-Src],} \\ \frac{d\text{[Cbp-Src]}}{dt}=kc\text{[Cbp][Src]}-k\text{\_}s\text{[Cbp-Src],} \end{matrix}$$

with the conservation laws $\text{[Cbp]}_{\text{tot}}=\text{[Cbp]}+\text{[Cbp-Src]}$, $\text{ [Src]}_{\text{tot}}=\text{[Src]}+\text{[Cbp-Src]}$.

$$\begin{matrix} \frac{d\text{[X}_{\text{in}}\text{]}}{dt}=-k_{out}\text{[X}_{\text{in}}\text{]}+k_{in}\text{[X}_{\text{out}}\text{],} \\ \frac{d\text{[X}_{\text{out}}\text{]}}{dt}=k_{out}\text{(Vr/Vn)[X}_{\text{in}}\text{]}-k_{in}\text{(Vr/Vn)[X}_{\text{out}}\text{],} \end{matrix}$$

with the conservation law $\text{[X]}_{\text{tot}}=\text{[X}_{\text{in}}\text{]Vr}+\text{[X}_{\text{out}}\text{]Vn}$. Here, Vr and Vn, which satisfy the relation Vr + Vn = 1, denote the raft volume ratio and the non-raft volume ratio, respectively.

The steady-state solution of these systems can be written as:

$$\text{[pSS]}=K_{dep}\frac{\text{[SS]}}{K_{M}+\text{[SS]}}\text{[SS]}_{\text{tot}},$$

where $K_{M}=(k\text{\_}s+kp)/ks$ and $K_{dep}=kd/kp$.

$$\begin{matrix} \text{[Cbp-Src]}=\frac{\text{[Src]}_{\text{tot}}+\text{[Cbp]}_{\text{tot}}+K_{dc}}{2} \\ +\frac{\sqrt{(\text{[Src]}_{\text{tot}}+\text{[Cbp]}_{\text{tot}}+K_{dc})^{2}-4\text{[Cbp]}_{\text{tot}}\text{[Src]}_{\text{tot}}}}{2}, \end{matrix}$$

where $K_{dc}=k\text{\_}c/kc$.

$\begin{matrix} \text{[X}_{\text{in}}\text{]}=\frac{k_{in}/k_{out}\text{(Vr/Vn)}}{1+k_{in}/k_{out}}\frac{\text{[X]}_{\text{tot}}}{\text{Vr}}, \\ \text{[X}_{\text{out}}\text{]}=\frac{1}{1+k_{in}/k_{out}\text{(Vr/Vn)}}\frac{\text{[X]}_{\text{tot}}}{\text{Vn}}. \end{matrix}$.
