# Supplementary material for: Roles of Raft-Anchored Adaptor Cbp/PAG1 in Spatial Regulation of c-Src Kinase
Source: PLoS One. 2014 Mar 27;9(3):e93470. doi: 10.1371/journal.pone.0093470 (PMC3968143; doi:10.1371/journal.pone.0093470)
Supplement: Text S2 — Random sampling for parameter searches. (DOCX) [file pone.0093470.s010.docx]

**Random sampling for parameter searches**

As shown in Figure 2, increases in c-Src and Cbp expression are relatively slow processes and thus phosphorylation events caused by c-Src kinase are often in a quasi-steady state. Therefore, we hereafter focused on the steady-state solution of the model. The full system is too complicated for an analytical study; however, subsystems (Figure S1) of the models can be solved, as shown in Text S1. This is helpful in estimating the steady-state solution of the full system; thus the solution may be written in the following form:

***F* = *F*([Src]_tot_, [Cbp]_tot_, [SS]_tot_, *K_M_, K_dep_, K_dc_, k_cin_/k_cout_, k_sin_/k_sout_, k_ssin_/k_ssout_*,*Vr/Vn*)**,

where ***F*** denotes an arbitrary function which consists of the stationary-state solution. The symbol **[∙∙∙]_tot_** denotes the total concentration of the molecules. The Michaelis-Menten constant ***K_M_*** is written as ***K_M_* = (*k_s* + *kp*)/*ks***. The constant ***K_dep_***, defined as ***K_dep_* = *kd*/*kp***, measures the rate of the dephosphorylation/phosphorylation cycle. ***K_dc_***, defined as ***K_dc_* = *k_c/kc***, is the equilibrium constant for the Cbp- and c-Src-binding reactions, where ***kc*** is the association rate constant and ***k_c*** is the dissociation rate constant.

Using the formula described in Text S1, we set the parameters controlling molecular localization in raft and non-raft fractions (***k_cin_, k_cout_, k_sin_, k_sout_, k_ssin_*** and ***k_ssout_***). The parameters for c-Src localization (***k_sin_*** and ***k_sout_***) were set so that c-Src is equally distributed in raft and non-raft fractions (Figure 2G, Table S2 and S3), while the parameters for FAK localization (***k_ssin_*** and ***k_ssout_***) were set so that FAK is exclusively localized in non-raft fractions (Figure 2G and 2H, Table S2 and S3). The parameters for Cbp localization were estimated from the results of the DRM separation experiments. The band intensities of DRM-Cbp and non-DRM-Cbp were estimated from 18 h treated Csk^-/-^ MEF / c-Src / p-BKT2-Cbp cells (Figure 2H), and the ratio of these two intensities was then calculated (Table S2 and S3).

To obtain a plausible parameter space that minimizes a target function, an optimization method was employed. A target function was chosen as the residual sum of squares (RSS) (Materials and methods). Since most of the parameters in the present system are unknown, performing a local parameter estimation would not lead to conclusive results. Therefore, a random sampling approach was employed to obtain a global description of the parameter space. Ten thousand parameter sets were randomly generated and the RSS for each parameter set was calculated, both in the sequestration model and the ternary model. Random parameter sets were generated from an exponential distribution bound to certain intervals (Table S1). To simplify the following analyses, values for **[FAK]_tot_**, ***kc*** and ***k_c*** were fixed, and ***ks1***, ***k_s1***, ***ks2***, ***k_s2***, ***kp1***, ***kp2***, and ***kd*** were varied (Table S2 and S3). The results of the random sampling are shown in Figure S2. The random sampling was performed in the two models independently. The two figures showed similar behavior, as the parameter space with RSS < 0.015 spreads to a wide range, and seems to satisfy the relation log ***K_M_*** = log ***K_dep_***. This might suggest that it is difficult to identify the global minimum of the parameter space. However, we confirmed that there were no significant differences in behaviors of the phosphorylation curves by selecting several good-fit parameter values satisfying RSS < 0.015. Therefore, the best-fit parameter (i.e., the parameter having the smallest RSS) was chosen. The phosphorylation curves obtained using the estimated parameter values are shown in Figure 3B. The estimated parameter values are shown in Tables S2 and S3.
